# Convergent Synthesis of Thioether Containing Peptides

**DOI:** 10.3390/molecules25010218

**Published:** 2020-01-05

**Authors:** Spyridon Mourtas, Christina Katakalou, Dimitrios Gatos, Kleomenis Barlos

**Affiliations:** 1 Department of Chemistry, University of Patras, 26510 Rio Patras, Greece; s.mourtas@upatras.gr (S.M.); c.katakalou@yahoo.gr (C.K.); d.gatos@upatras.gr (D.G.); 2 Institute of Chemical Engineering Sciences of the Foundation for Research and Technology Hellas (FORTH/IEC-HT), 26504 Rio Patras, Greece; 3 CBL-Patras, Patras Industrial Area, Block 1, 25018 Patras, Greece

**Keywords:** haloacylated peptides, aminothiols, bis(aminoalkyl)dithiols, convergent solid phase peptide synthesis, trityl-type resins

## Abstract

Thioether containing peptides were obtained following three synthetic routes. In route A, halo acids esterified on 2-chlorotrityl(Cltr) resin were reacted with *N*-fluorenylmethoxycarbonyl (Fmoc) aminothiols. These were either cleaved from the resin to the corresponding (Fmoc-aminothiol)carboxylic acids, which were used as key building blocks in solid phase peptide synthesis (SPPS), or the *N*-Fmoc group was deprotected and peptide chains were elongated by standard SPPS. The obtained *N*-Fmoc protected thioether containing peptides were then condensed either in solution, or on solid support, with the appropriate amino components of peptides. In route B, the thioether containing peptides were obtained by the reaction of *N*-Fmoc aminothiols with bromoacetylated peptides, which were synthesized on Cltr-resin, followed by removal of the *N*-Fmoc group and subsequent peptide elongation by standard SPPS. In route C, the thioether containing peptides were obtained by the condensation of a haloacylated peptide synthesized on Cltr-resin and a thiol-peptide synthesized either on 4-methoxytrityl(Mmt) or trityl(Trt) resin.

## 1. Introduction

**Figure 1:**
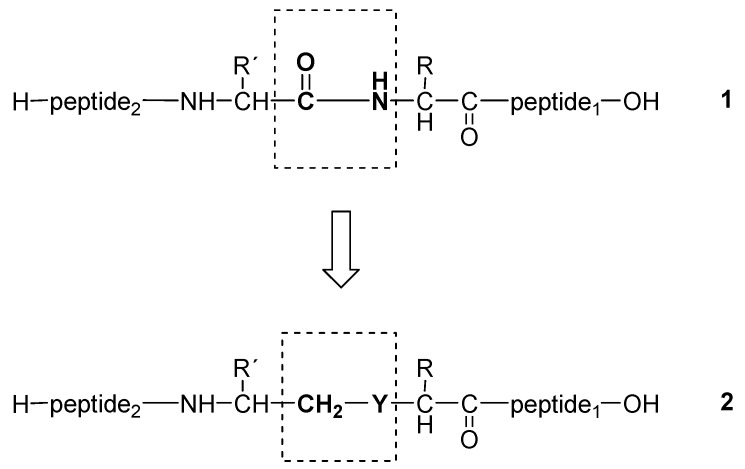
Peptide bond isosteres: Y = CH_2_ (a), NH (b), O (c) and S (d).

Peptides are used as therapeutic agents for controlling diseases related to peptide functions. However, the use of native peptides for clinical applications has been hampered mainly by their rapid degradation by proteases, poor oral bioavailability, difficult transportation through cell membranes and nonselective receptor binding [1]. These limitations of peptides have led to the synthesis of peptidomimetics through numerous modifications of peptide structures [2,3,4]. Among the several peptide modifications, isosteric replacement of a peptide bond represents a viable approach in the rational design of peptidomimetics. Peptidomimetics in which the peptide amide bond is replaced by ethylene (**1a**)-, methyleneimino (**1b**)-, methyleneoxy (**1c**)- and methylenethio (**1d**)-groups (Figure 1) were found to exhibit potential pharmaceutical application [4,5,6,7,8,9,10,11,12,13,14,15]. Such modifications alter the physical and chemical properties of the native peptide, reducing its peptide character and leading to peptide analogues with increased resistance to proteolytic enzymes, flexibility and lipophilicity, while they also confer diverse electrostatic properties and new secondary conformations on the peptidomimetic chain, often resulting in improved pharmacokinetic properties [2,16,17].

Solid-phase synthesis (SPS) is a common technique for peptide synthesis. The classical step-by-step solid-phase peptide synthesis (SPPS) is a well-established methodology for the synthesis of small to medium-sized peptides [18]. Besides SPPS, the convergent solid-phase peptide synthesis (CSPPS) has been developed for the preparation of larger and/or complex peptides and proteins [19,20,21,22,23,24], based on the fact that no directional restrictions exist in CSPPS and the chain elongation can be performed with equal possibility of success to any direction. CSPPS methods include fragment condensation such as the solid- or solution-phase based protocols. Both methods consist of the rational and retro-synthetic detachment of segments in the native sequence, which in a synthetic flow would be connected in the appropriate manner in the solid- or in the solution-phase.

The very acid labile 2-chlorotrityl (Cltr) resin [25] and the Fmoc/^t^Bu methodology [23,26] have been developed and widely used for SPPS and CSPPS of protected peptides. In general the sequential condensation of fluorenylmethoxycarbonyl (Fmoc)/tert-butyl (^t^Bu) protected peptide fragments on a solid support to larger peptides (convergent synthesis, solid phase fragment condensation (SPFC)) is a simple method for the preparation of large or difficult peptides by convergent methods [27,28]. The main limitation of this method is that, in some cases, the *N*-Fmoc deprotection of the resin-bound peptide and the subsequent condensation with the next fragment proceeds slowly and incomplete. A solution to this problem is provided with the cleavage of the protected peptide from the resin in order to perform the *N*-Fmoc removal and the condensation in solution. This type of condensation is also advantageous from an economical point of view, since the C- and *N*-components are used in equimolar amounts. The key step of this method is the esterification of the protected peptide, which is cleaved from the resin, at the C-terminal carboxyl function, and for this reason, the usefulness of 2-chlorotrityl chloride monomer as a temporary carboxy-protecting group has been studied in CSPPS [24].

In this manuscript, we describe our efforts in the synthesis of peptidomimetics of type **2** (Figure 1), by using simple methods of CSPPS. In particular, we describe three methods in the convergent synthesis of type **2d** peptide isosteres, in which the amide bond is replaced by CH_2_-S isostere bond (thioether bond), using methods of solid phase peptide fragment condensation or solution fragment condensation through the formation of an amide bond, and the condensation of two fragments through the formation of a thioether bond.

## 2. Results and Discussion

**Scheme 1:**
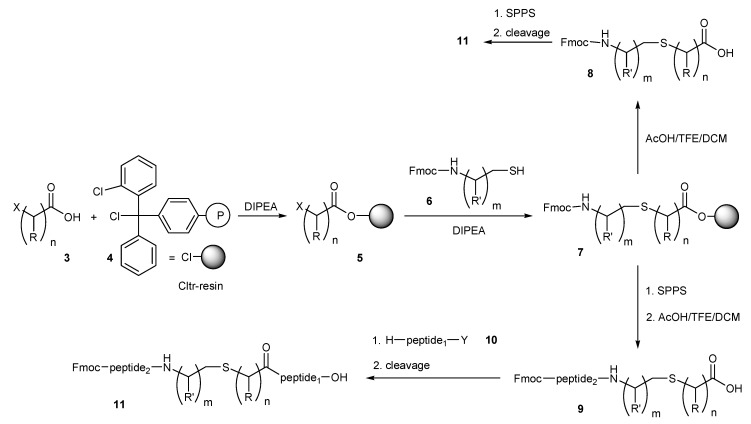
Route A for convergent solid-phase peptide synthesis (CSPPS) of thioether containing peptides. Halo acids esterified on Cltr-resin were reacted with *N*-Fmoc aminothiols and peptide chains were elongated either by direct SPPS or CSPPS, or cleavage of the (Fmoc-aminothiol)carboxylic acids and their use in SPPS; R = H (n = 1–5), CH_3_ (n = 1); R′ = H (m = 1–5), CH_3_, CH(CH_3_)_2_, CH_2_CH(CH_3_)_2_, CH(CH_3_)CH_2_CH_3_, CH_2_(C_6_H_5_) (m = 1); X = Cl, Br; Y = *O*-Cltr monomer (**10a**), *O*-Cltr-resin (**10b**).

In an effort to apply standard methods of convergent solid phase peptide synthesis (CSPPS) [21,24,26] in the preparation of the thioether containing peptides **11** (Scheme 1), we esterified the halo acids **3** with 2-chlorotrityl(Cltr) resin **4** in dichloromethane (DCM) using *N*,*N*-diisopropylethylamine (DIPEA) as the hydrogen halide acceptor. The reaction proceeds fast independently from the bulkiness of **3** and resins containing 0.6–1.0 mmol **3**/g were obtained. The loading of the resins **5** was determined by Gas Chromatography (GC) after cleavage of the haloacids by treatment of resin probes with 3% trifluoroacetic acid (TFA) in DCM for 30 min at room temperature. The obtained haloacid derivatives **5** were further reacted with a two-fold molar excess of the *N*-fluorenylmethoxycarbonyl (Fmoc) aminothiols **6** [29] and DIPEA in dimethylformamide (DMF) for 1–12 h at room temperature. Especially the more electrophilic α-haloacids, such as the bromoacetic acid, react very fast with the less hindered Fmoc-aminothiols **6** (in less than 30 min) to the resin-bound (*N*-Fmoc aminothiol)carboxylic ester **7**. Treatment of **7** with the cleavage mixture acetic acid (AcOH)/trifluoroethanol (TFE)/dichloromethane (DCM) (1:2:7) for 30 min at room temperature followed by extractive work up gave the key building blocks **8**, which were applied in usual step by step solid phase peptide synthesis (SPPS), by Fmoc/tert-butyl (^t^Bu) methodology leading to the target peptides **11**. Instead of using **8** in SPPS, direct peptide elongation on resin **7**, by standard SPPS and cleavage from Cltr-resin by the cleavage mixture AcOH/TFE/DCM (1:2:7) gave the thiol-containing peptide **9**. The obtained segment **9** can be condensed with the amino components of the peptide fragment **10** either in solution (in case of *O*-Cltr protected peptide fragment **10a**) [24] by using solution fragment condensation strategy, or in solid phase (in case of resin-bound peptide fragments **10b**). In the first case, fragment condensation of **9** with the *O*-Cltr protected monomer **10a** requires an equimolar amount of **9**, while in the second case condensation of **9** with the *N*-free amino group of peptides attached on the Cltr-resin **10b** requires a two-fold molar excess of **9** to be completed.

**Scheme 2:**
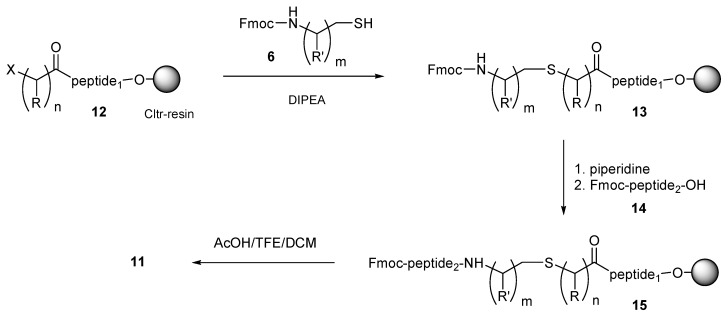
Route B for CSPPS of thioether containing peptides. The thioether containing peptides were obtained by the reaction of *N*-Fmoc aminothiols with bromoacetylated peptides, which were synthesized on Cltr resin, followed by removal of the *N*-Fmoc group and peptide elongation; R = H (n = 1–5), CH_3_ (n = 1); R′ = H (m = 1–5), CH_3_, CH(CH_3_)_2_, CH_2_CH(CH_3_)_2_, CH(CH_3_)CH_2_CH_3_, CH_2_(C_6_H_5_) (m = 1); X = Cl, Br.

By applying a similar method, esterification of the halo acid **3** with the *N*-free amino group of peptides elongated on Cltr-resin gave the resin-bound haloacylated peptide **12** (Scheme 2). For this synthesis step by step SPPS (Fmoc/^t^Bu methodology) was used. The resin-bound haloacylated peptide **12** was then condensed with a two-fold molar excess of the *N*-Fmoc aminothiol **6** and DIPEA in DMF for 1–12 h at room temperature to the resin-bound peptide **13**. Removal of the *N*-Fmoc group by piperidine liberated the amino group of the peptide chain, enabling the peptide elongation either by standard SPPS or by CSPPS using the *N*-Fmoc protected peptide fragment **14**. The obtained resin-bound thiol-containing peptide **15** was finally treated with the cleavage mixture AcOH/TFE/DCM (1:2:7) to the thioether containing peptide **11**.

**Figure 2:**
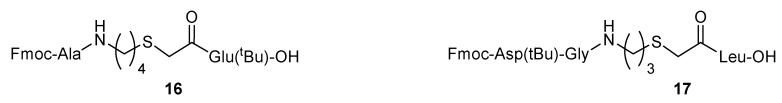
Structure of thioether containing peptide **16** (synthesized by using route A), and **17** (synthesized by using route B).

In order to evaluate the applicability of the above described methods in SPPS, we synthesized the simple thiolether containing peptide **16** (by using route A) and **17** (by using route B; Figure 2). Their synthesis was done on Cltr-resin by using Fmoc/^t^Bu strategy, according to Scheme 1 and Scheme 2, respectively.

In brief, amino acid couplings were achieved by using 1-hydroxybenzotriazole/*N*,*N*′-diisopropylcarbodiimide (HOBt/DIC) in *N*-methyl-2-pyrrolidone (NMP). As the *N*-Fmoc aminothiols **6**, the corresponding *N*-Fmoc aminobutanethiol and *N*-Fmoc aminopropanethiol derivatives were used. Both thioether containing peptides (**16** and **17**) were prepared in high yield (87 and 90% yield, respectively) and purity (95 and 98%, respectively) as confirmed by hplc analysis (see Figure S1 in Supplementary Materials section), revealing that these methods could be used for the preparation of larger thioether containing peptides. The correct mass of peptide analogues **16** and **17** was determined by ESI-MS analysis (**16**: [M + H] calc.: 641.28; found: 641.42 and **17**: [M + H] calc.: 712.31; found: 712.56). In this method, although the thioether formation between the *N*-Fmoc aminothiol **6** and the haloacylated peptide **12** proceeds relatively fast, the fragment coupling of the resin-bound *N*-Fmoc deprotected thiol-peptide **13** with the *N*-Fmoc peptide fragment **14**, proceeds slowly (6–12 h) and in some cases is incomplete, similarly to route A.

Since the condensation of the different fragments in both routes A (either by using **8** in SPPS, or by fragment coupling between **9** and **10**) and B (fragment coupling between the *N*-Fmoc deprotected **13** and **14**) proceeds through amide formation, which is relatively slow, we proposed a third method (route C), for the synthesis of thioether containing peptides **11**, which enables the fast condensation reaction between two peptide fragments.

**Scheme 3:**
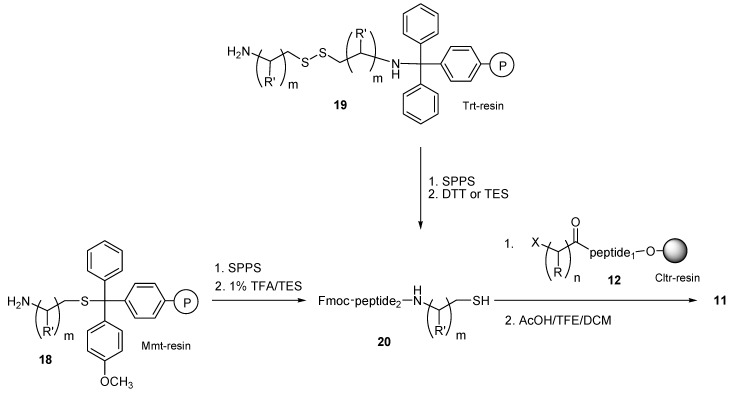
Route C for CSPPS of thioether containing peptides. The thioether containing peptides were obtained by the condensation of a haloacylated peptide which was synthesized on Cltr-resin and a thiol-peptide which was synthesized either on Mmt-resin, or on Trt-resin; R = H (n = 1–5), CH_3_ (n = 1); R′ = H (m = 1–5), CH_3_, CH(CH_3_)_2_, CH_2_CH(CH_3_)_2_, CH(CH_3_)CH_2_CH_3_, CH_2_(C_6_H_5_) (m = 1); X = Cl, Br.

In this method (route C), the synthesis of thioether containing peptides **11** does not proceed through amide bond formation (as in routes A and B), but through thioether bond formation, by the nucleophilic attack of the *N*-Fmoc protected thiol-peptide **20** with the resin-bound bromoacetylated peptide **12** (Scheme 3). The first step in this procedure is the preparation of the thiol-peptide **20**. For this, we used either aminothiols attached on 4-methoxytrityl(Mmt) resin through their thiol function **18** [29], or resin-bound bis(aminoalkyl)dithiol attached on trityl(Trt) resin through their free amino group **19**. Peptide elongation was achieved by using standard SPPS (Fmoc/^t^Bu strategy), while amino acid coupling was achieved by using HOBt/DIC in NMP. The derived resin bound peptides were then quantitatively cleaved with 1% TFA in presence of DCM/triethylsilane (TES) (95:5) (in case of peptides elongated on resins **18**), and dithiothreitol (DTT) or TES (in case of peptides elongated on resins **19**), to the *N*-Fmoc thiol-peptides **20** (Scheme 3). In the next step of the synthesis, the *N*-Fmoc protected thiol-peptide fragments **20** were condensed with the resin-bound halo acylated peptides **12** in DMF and DIPEA and the obtained resin-bound thioether peptides were completely cleaved from the Cltr-resin by treatment with AcOH/TFE/DCM (1:2:7) for 15 min at room temperature. It was noticed that the condensation between the bromoacetylated peptide segments **12** and the *N*-Fmoc thiol-peptides **20** proceeded with exceptional ease and in some cases very fast (10 min), as it is shown below, in the example of choice (thioether containing peptide **26**).

Although we have already published the advantages of resin bound aminothiols **18** (loaded through their thiol group on Mmt-resins) in the synthesis of *N*-Fmoc thiol-containing peptides [29], the use of bis(aminoalkyl)disulfides bound on Trt-resins **19** (through their amine group) in SPPS is described for the first time. These resins may offer several advantages in SPS, SPPS and CSPPS, based on their ability to form either thiol-peptides by reductive cleavage, or bis(aminoalkyl)thiol peptides by treatment of the resin with 1% TFA in presence of DCM/TES (95:5). The first case (reductive cleavage of thiol-peptides **20** from resins **19**) can be used for the synthesis of the *N*-Fmoc protected thiol-peptides **20**, by their treatment with DTT or TES, which are considered as mild reducing agents. Thus, in an effort to optimize the cleavage of the thioether containing peptides **11** synthesized on Trt-resins **19**, we compared the use of DTT or TES in terms of yield and purity. For this, the resin-bound bis(aminoalkyl)dithiol derivative (R′ = CH_3_) **19** was coupled with *N*-Fmoc-leucine by using HOBt/DIC as the carboxylic acid activator. The derived resin was treated with a 10 fold molar excess of DTT or TES. In both cases the expected Fmoc-Leu-alaninothiol was completely cleaved from the resin after 12 h reaction at room temperature. Hplc analysis of the crude product showed that the use of TES is superior in terms of product purity, as a purity of 67% was noticed for the crude product of Fmoc-Leu-alaninothiol cleaved by DTT, while a purity of 98% was noticed for Fmoc-Leu-alaninothiol cleaved by TES (see Figure S2 in Supplementary Materials section). The correct mass of the released Fmoc-Leu-alaninothiol was determined by ESI-MS analysis ([M + H] calc.: 427.21; found: 427.32). For this reason, TES was used for the cleavage of the more complicated peptides that were prepared by this route.

**Figure 3:**
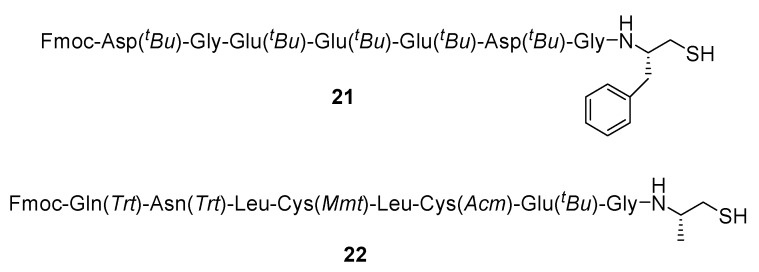
Structure of thiol-peptides **21** (ProTa (69-75) derivative) and **22** (Hirudine (11-18) derivative), synthesized on Trt-resins **19** (R′ = CH_3_ (**21**); –CH_2_–Ph (**22**)).

As an example of the more complicated thiol-containing peptides that we prepared by the use of resin **19**, we describe the synthesis of two thiol-peptide analogues: **21** (Figure 3; synthesized on Trt-resin **19** where R′ = CH_3_) and **22** (Figure 3; synthesized on Trt-resin **19** where R′ = –CH_2_–Ph).

The first product (peptide structure **21**) is a thiol derivative of the prothymosin a peptide sequence (ProTa (69-75)) prepared in high yield (82%) and purity (>98%), as can be seen by the hplc analysis of the crude product (see Figure S3A in Supplementary Materials section). Its correct mass was determined by ESI-MS analysis (see Figure S3B in Supplementary Materials section): ([M + H] calc.: 1401.69; found: 1401.59). The second product (peptide structure **22**) is a peptide derivative of hirudine (Hir (11-18)), which contains two differently protected cysteines: Cys14(*Mmt*) and Cys16*(Acm)*. By using this resin, we were able to synthesize the thiol-peptide **22**, after its cleavage from the Trt resin **19** by TES, without affecting the Cys14*(Mmt)* protecting group. This product was also prepared in high yield (78%) and purity (90%), as can be seen by the hplc analysis (see Figure S3C in Supplementary Materials section), while its correct mass was determined by ESI-MS: ([M + 2H] calc.: 1029.46; found: 1029.86 (see Figure S3D in Supplementary Materials section)).

**Scheme 4:**
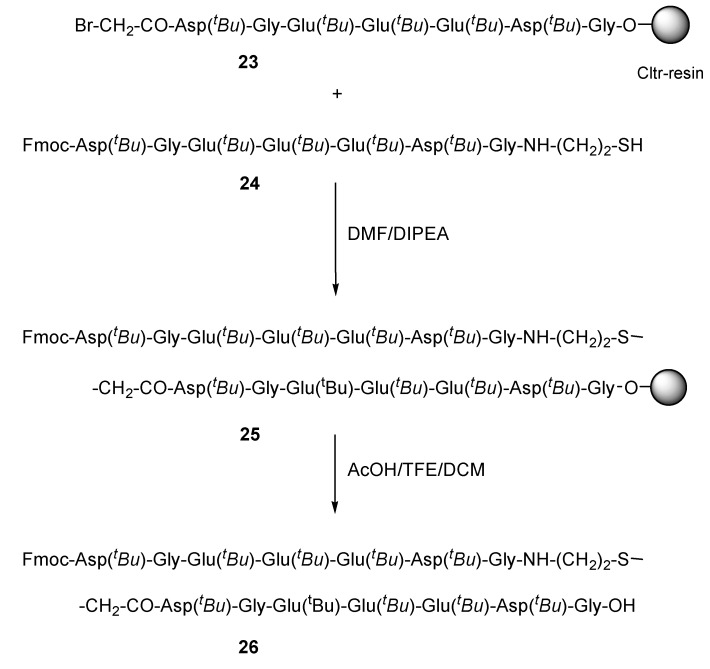
Synthetic procedure of **26** by using route C.

As an example of the final step of our strategy for the synthesis of thioether containing peptides **11** by route C, we present the synthesis of the cysteamine derivative **26**, which is composed by two prothymosin a segments (ProTa (69-75)) linked by a thioether bond (Scheme 4). For this synthesis, the resin-bound bromoacetylated peptide **23** was initially prepared on Cltr-resin by using standard SPPS methods (Fmoc/^t^Bu method and HOBt/DIC for the amino acid activation). Then a two-fold molar excess of **24** was condensed with **23** in DMF/DIPEA for 10 min at room temperature (Scheme 4). The resin **25** was treated with AcOH/TFE/DCM (1:2:7) and the protected peptide **26** was obtained in 90% yield and 92% purity according to HPLC analysis (see Figure S4 in Supplementary Materials section). The correct mass of the obtained peptide **26** was determined by ES-MS analysis ([M + 2H] calc.: 1191.60; found: 1191.80).

**Scheme 5:**
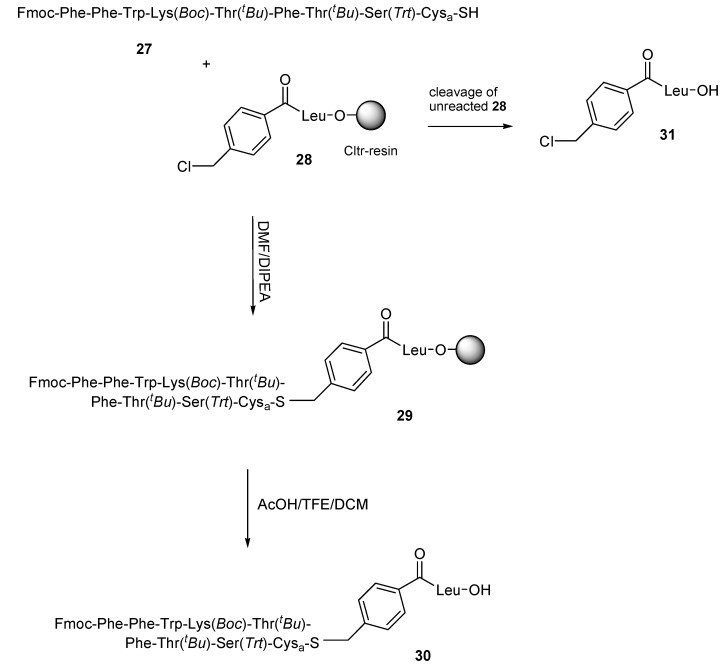
Synthetic procedure of **30** by using route C. The reaction process was monitored by hplc analysis following the peaks of the desired product **30** and un-reacted **31** after their cleavage from the resin (see Figure S5 in Supplementary Materials section).

Ligation strategies on haloacylated peptides have been found to proceed very fast in case of bromoacylated peptides, while in case of chloroacylated peptides moderate reactivities were found [30,31]. In order to examine this parameter, we planned the fragment condensation of thiol-peptides to chloroacylated peptides. As an example we synthesized the somatostatin analogue (6-14) where Cys14 was replaced by cysteamine (Cys_a_; 2-aminoethanethiol) **27**. In this experiment, the thiol-peptide **27** contains the acid sensitive Ser*(Trt)* (*O*-Trt is cleaved by 1% TFA) and for this reason it was synthesized on Trt-resin **19**. The thiol-peptide **27** was condensed with the 4-chloromethylbenzoyl-Leu-O-Cltr resin **28** (Scheme 5) in a 1.5:1 molar ratio in DMF and DIPEA and the reaction process was followed by hplc analysis, after the treatment of resin probes with AcOH/TFE/DCM (1:2:7) for 15 min at room temperature, by which the desired product **30** and the un-reacted **31** were identified. Analysis of the hplc chromatograms (see Figure S5 in Supplementary Materials section) showed a high percentage of un-reacted **31** (absorbance ratios of **30/31**: 46/54) after 2 h reaction time, due to incomplete reaction of **27** with **28**, while a significant percentage of **31** (absorbance ratios of **30/31**: 82/18) was still observed even after 24 h reaction. It should be noted that, although the reaction progress was rather slow, no significant by-products were detected during the extended reaction time. Product **30** was identified by ESI-MS ([M − Trt + 2H] calc.: 902.46; found: 903.38; (see Figure S5 in Supplementary Materials section).

**Scheme 6:**
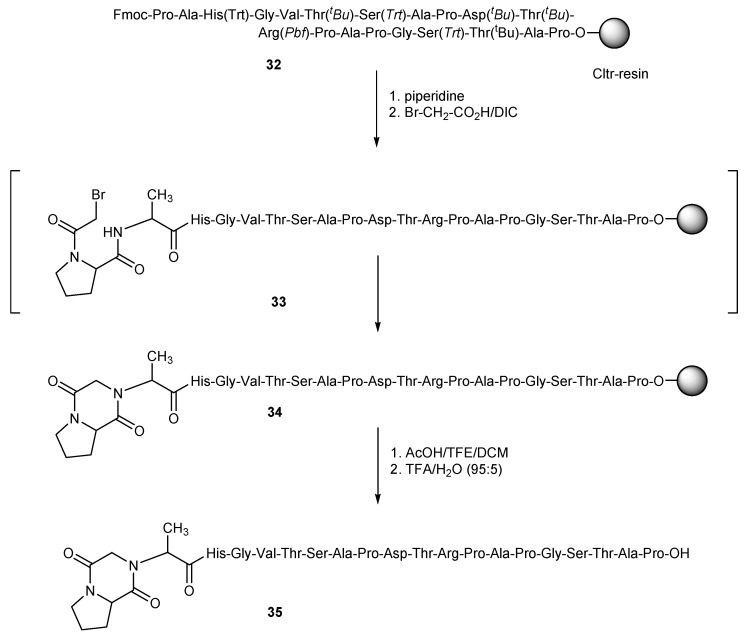
Diketopiperazine peptide derivative **35** formation of bromoacetylated-MUC-1 peptide.

In case of haloacetyl-peptides, which contain various strong nucleophiles, one should be aware of possible side reactions of the nucleophiles with the haloacetyl moiety. This is a well-known problem especially for bromoacetylated-methionine (Met)-peptides [31,32,33]. In this work we found similar instability issues for bromoacetylated-proline (Pro)-peptides. As an example, resin-bound MUC-1 **32** was treated with piperidine to liberate the *N*-terminus of the peptide sequence and this was reacted with a three-fold molar excess bromoacetic acid and DIC in NMP (Scheme 6). After cleavage of the obtained peptide from the resin and deprotection with TFA/water (95:5) for 3 h at room temperature, the main product of the synthesis, instead of the expected haloacylated peptide, was a product with a molecular mass [M − 81] ([M − 81 + 2H]: calc.: 963.97; found: 964.09). This corresponds to a peptide with one less HBr, which was attributed to the diketopiperazine peptide derivative **35**, obviously prepared by the nucleophilic attack of the *N*-terminus group of alanine to the carbon atom that bears the bromine. This nucleophilic attack is possibly favored by the proximity of these atoms, which is due to the presence of proline just before the haloacid in the peptide chain (Scheme 6).

## 3. Materials and Methods

### 3.1. Materials

All chemicals were purchased from Sigma-Aldrich OM, Athens, Greece, except 2-Chlorotrityl polystyrene (Cltr) resin and Fmoc-protected amino acids, which were gifted from CBL Patras S.A. (Industrial area of Patras, Building block 1, GR-25018, Patras, Greece). All chemicals were used without further purification, according to the manufacturer’s instructions and safety precautions. TFA was used in a properly ventilated hood, wearing protective gloves/protective clothing/eye protection/face protection.

### 3.2. Analytical Methods

Thin layer chromatography (TLC) was performed on precoated silica gel 60 F254 plates (Merck, Darmstadt, Germany) and spot detection was carried out by UV light, or by charring with a ninhydrin solution. HPLC analysis was performed on a Waters 600E multisolvent delivery system (Milford, MA, USA), combined with Waters 991 photodiode array detector, using a Nucleosil C8 (4 mm × 125 mm, 7 μm) and a linear gradient from 50 to 100% acetonitrile (containing 0.1% TFA) in water (containing 0.1% TFA) within 30 min at a flow rate of 1 mL/min or Lichroshere RP-8 (4 mm × 150 mm, 5 μm) and a linear gradient from 20 to 100% acetonitrile (containing 0.1% TFA) in water (containing 0.1% TFA) within 30 min at a flow rate of 1 mL/min. Chromatograms were detected at 265 nm. ES-MS spectra were recorded on a Micromass Platform L.C. (Manchester, UK) at 30 V.

### 3.3. Synthetic Procedures

#### 3.3.1. General Protocol for the Synthesis of Bis(aminoalkyl)dithiols

In a solution containing *S-*trityl (Trt) aminothiols [29] (5.8 mmol, R′ = H, m = 1: 1.85 g; H, m = 2: 1.93 g; H, m = 3: 2.02 g; H, m = 4: 2.10 g; H, m = 5: 2.18 g; CH_3_, m = 1: 1.93 g; CH(CH_3_)_2_, m = 1: 2.10 g; CH_2_CH(CH_3_)_2_, m = 1: 2.18 g; CH(CH_3_)CH_2_CH_3_, m = 1: 2.18 g; CH_2_(C_6_H_5_) and m = 1: 2.38 g) in tetrahydrofuran (THF; 9 mL), 30% (*w*/*w*) hydrogen peroxide (H_2_O_2_) was added, drop-by-drop, at room temperature, until the formation of a white solid (6.0 mmol; 0.47 mL). The reaction mixture was left for about 1 h at room temperature and then condensed under reduced pressure. The oily product was suspended in diethyl ether (DEE) and subjected to extraction in DEE/water. The organic phase was washed twice with water and then dried over Na_2_SO_4_, filtered and condensed. The oily product was dissolved in 4 mL of dichloromethane (DCM) and to this solution a mixture of 10% trifluoroacetic acid (TFA) in DCM (4 mL) was slowly added, and the reaction mixture was stirred for 1 h at room temperature. The solvent was condensed under reduced pressure and then re-dissolved in THF, converted to the corresponding hydrochloric salt and precipitated in ethanol (EtOH)/1,4-Dioxane 4:1. The solid was filtered, washed and then dried in vacuo (25 °C/38 mmHg) over KOH. The yield (%) of each product was as following: R′ = H, m = 1: 75%; H, m = 2: 74%; H, m = 3: 74%; H, m = 4: 74%; H, m = 5: 72%; CH_3_, m = 1: 70%; CH(CH_3_)_2_, m = 1: 72%; CH_2_CH(CH_3_)_2_, m = 1: 72%; CH(CH_3_)CH_2_CH_3_, m = 1: 70%; CH_2_(C_6_H_5_) and m = 1: 70%.

#### 3.3.2. General Protocol for the Synthesis of Resin-bound Bis(aminoalkyl)dithiols

Trityl(Trt) resin (0.3 mmol, 0.3 g) was suspended in a plastic syringe equipped with porous polypropylene frit and washed with DCM (3 min × 1 min) and bis(aminoalkyl)dithiol (0.6 mmol, R′ = H, m = 1: 0.19 g; H, m = 2: 0.20 g; H, m = 3: 0.21 g; H, m = 4: 0.22 g; H, m = 5: 0.26 g; CH_3_, m = 1: 0.20 g; CH(CH_3_)_2_, m = 1: 0.22 g; CH_2_CH(CH_3_)_2_, m = 1: 0.23 g; CH(CH_3_)CH_2_CH_3_, m = 1: 0.23 g; CH_2_(C_6_H_5_) and m = 1: 0.25 g) dissolved in 3 mL of DCM and *N*,*N*-diisopropylethylamine (DIPEA; 1.2 mmol, 0.21 mL) was added and the resin was shaken for 3 h at room temperature. DCM was filtered and the resin was washed with DCM (1 min × 2 min), DCM/methanol (MeOH)/DIPEA (85:10:5; 3 min × 15 min), *N*-methyl-2-pyrrolidone (NMP; 5 min × 2 min), isopropyl alcohol (iPrOH; 2 min × 5 min) and DEE (4 min × 2 min). The final resin was dried by suction in air for 15 min and further dried for 24 h in vacuo (25 °C/0.5 mmHg) over KOH and stored at 4 °C.

#### 3.3.3. Solid-Phase Peptide Assembly, General Protocol

Solid-phase peptide synthesis was carried out manually in plastic syringes equipped with porous polypropylene frits at room temperature, using the fluorenylmethoxycarbonyl (Fmoc)/tert-butyl (^t^Bu) strategy [21,23].

##### Pre-Activation of Fmoc-Amino Acids and (Fmoc-Aminothiol)Carboxylic Acids

The Fmoc-amino acid or the (Fmoc-aminothiol)carboxylic acid (4.5 mmol) and 1-hydroxybenzotriazole hydrate (HOBt; 5.8 mmol) were dissolved in NMP (4.5 mL) and cooled to 5 °C. Then, diisopropylcarbodiimide (DIC; 5.0 mmol) was added and the mixture was stirred for 15 min at 5 °C.

##### Coupling of Fmoc-Amino Acids and (Fmoc-Aminothiol)Carboxylic Acids

The solution of the pre-activated amino acid (the above paragraph) was added to the resin-bound amino-acid or peptide (5.0 g, loading 0.3 mmol/g) and was shaken for 3 h at room temperature. A sample was taken to check the reaction completion by Kaiser test. In case of incomplete coupling (positive Kaiser test) recoupling was performed with a fresh solution of activated amino acid (6 mmol).

##### Fmoc-Group Removal and Fmoc-Removal Test

The resin-bound Fmoc-protected amino acids or peptides were treated twice with 25% piperidine in NMP (25 mL) for 30 min at room temperature. To check the completion of the *N*-Fmoc removal, 25% piperidine in NMP (20 μL) was added to a resin probe (approx. 2 mg) and the mixture was heated for 5 min at 100 °C. From the resulting solution 10 μL were spotted on a TLC plate and checked under a UV-lamp for UV-absorbing material. Alternatively, the solution was injected on HPLC and the Fmoc-material produced and released into solution was quantified at 265 nm. If the *N*-Fmoc removal test remained positive (violet spot under UV) the piperidine treatment was prolonged or repeated until achieving negative test. The resin was filtered and washed with NMP (×5), DCM (×3), DEE (×2) and dried in vacuo (25 °C/0.5 mmHg), otherwise it was used directly to the next step.

#### 3.3.4. General Procedure for the Loading of 2-Chlorotrityl(Cltr) Resin with Halo Acids

To a suspension of 1 g Cltr resin (1.5–1.9 mmol chloride/gr resin) in 10 mL of DCM, DIPEA (2 mmol) and the halo acid (1.5 mmol) were added. The resulting mixture was left to react for 20 min at 25 °C. The resin was filtered, suspended in 20 mL of a DCM/MeOH/DIPEA (80:15:5) mixture and was shaken for 1 h at 25 °C. The resin was filtered and washed once more with the above mixture, and then with NMP (5 mL × 10 mL), DCM (3 mL × 10 mL), diethylether (DEE; 3 mL × 5 mL) and dried in vacuo (25 °C/0.5 mmHg), otherwise it was used directly to the next step. The loading of the resins was determined by GC analysis after cleavage of the haloacids by treatment of resin probes with 3% trifluoroacetic acid (TFA) in DCM for 30 min at 25 °C.

#### 3.3.5. General Procedure for the Synthesis of Resin-Bound Haloacylated Peptides

Halo acid (three-fold molar excess over the resin bound peptide) was dissolved in NMP. The solution was cooled on ice bath and then DIC (3 mol equiv.) was added. The activated halo acid was added to the resin-bound peptide and allowed to react for 1–3 h at 25 °C. The resin was filtered and washed with NMP (×5), DCM (×3), DEE (×2) and dried in vacuo (25 °C/0.5 mmHg), otherwise it was used directly to the next step.

#### 3.3.6. General Procedure for the Condensation of *N*-Fmoc Aminothiols or *N*-Fmoc Thiol-Peptides with Resin-Bound Haloacids

The *N*-Fmoc aminothiol (two-fold molar excess over the resin-bound haloacid) or the *N*-Fmoc thiol-peptide (two-fold molar excess over the resin-bound haloacylated peptide fragment) was dissolved in the highest possible concentration in NMP and DIPEA (2.2 equiv.). This solution was added to the dry resin without pre-swelling and the reaction was left for 1–12 h at room temperature. The resin was filtered and washed with NMP (×5), DCM (×3), DEE (×2) and dried in vacuo (25 °C/0.5 mmHg), otherwise it was used directly to the next step.

#### 3.3.7. General Procedure for the Cleavage of Thiol-Peptides from Trityl(Trt) Resin

##### Method A:

Resin-bound bis(aminoalkyl)dithiol-peptides (0.2 mmol) were treated with DTT (10 equiv.) in NMP for 12 h at room temperature. The resin was filtered and washed twice with NMP and then subjected to an extraction process in DEE/H_2_O. The organic layer was collected, dried over Na_2_SO_4_, filtered and condensed under reduced pressure.

##### Method B:

Resin-bound bis(aminoalkyl)dithiol-peptides (0.2 mmol) were treated with TES (10 equiv.) in DCM for 12 h at room temperature. The resin was filtered and washed twice with DCM. The combined filtrates were concentrated on a rotary evaporator and to the residue DEE was added. The resulting solid was filtered, washed with DEE (×4) and dried in vacuo (25 °C/0.5 mmHg).

#### 3.3.8. General Procedure for the Cleavage of Thiol-Peptides from 4-Methoxytrityl(Mmt) Resin

A cleavage solution of 1.1% TFA in DCM/TES (95:5) (7 mL) was added on 0.5 g of resin and the mixture was stirred magnetically at room temperature. The resin was filtered and washed twice with 5 mL of the cleavage solution. The combined filtrates were concentrated on a rotary evaporator and to the residue DEE was added. The resulting solid was filtered, washed with DEE (×4) and dried in vacuo (25 °C/0.5 mmHg).

#### 3.3.9. General Procedure for the Cleavage of Peptides and Thioether Containing Peptide Fragments from 2-Chlorotrityl(Cltr) Resin

A cleavage solution of 1.1% TFA in DCM/TES (95:5) (7 mL) was added on 0.5 g of resin and the mixture was stirred magnetically at room temperature. The resin was filtered and washed twice with 5 mL of the cleavage solution. The combined filtrates were concentrated on a rotary evaporator and to the residue DEE was added. The resulting solid was filtered, washed with DEE (×4) and dried in vacuo (25 °C/0.5 mmHg).

## 4. Conclusions

Besides classical step-by-step synthesis, the convergent solid phase peptide synthesis (CSPPS) was used for the preparation of complex and difficult peptides and small proteins.

Peptides where the amide bond is replaced by its CH_2_–S isostere (thioether containing peptides) are very important peptide mimetics. Thus, we were interested in the synthesis of thioether containing peptides by convergent methods.

Comparing the methods discussed, route C (Scheme 3) offers significant advantages, over routes A and B, for the preparation of thioether containing peptides, mainly because the condensation of the thiol-peptide fragment **20** with the resin-bound bromoacetyl peptide **12** proceeds faster than: (i) the use of the thioether containing building block **8** in SPPS (Scheme 1; route A), or (ii) the condensation of the thioether containing peptide **9** with the peptide fragment **10** (Scheme 1; route A) or (iii) the condensation of the thiol-containing peptide fragment **14** with the peptide fragment **15** (Scheme 2; route B).

Regarding the synthesis of the thiol-peptide fragment **20**, in case that acid sensitive (cleaved under acidic solutions ≤1% TFA) amino acid side chain protecting groups are needed in the peptide sequence, we suggest its synthesis through reductive cleavage from resin **19**, instead of its acidic cleavage from resin **18** (by which the acid labile protecting group would be cleaved).

In contrary, routes B and C, which were based on the synthesis of the resin-bound haloacylated peptide **12**, have to be avoided when the sequence of **12** contains various strong nucleophiles, which may react with the haloacetyl moiety. This is a well-known problem for bromoacetylated-Met-peptides and we proved that bromoacetylated-Pro-peptides also suffered from instability issues, by their conversion to the corresponding diketopiperazine peptide derivatives. In this case, route A should be preferred, as in this route the thioether formation is performed on the resin-bound esterified haloacid **5**, thus, amino acids have not yet been introduced into the peptide sequence, avoiding the nucleophilic attack on the haloacetyl moiety by the nucleophilic groups that are present in the peptide chain of routes B and C.

## Supplementary Materials

The following are available online, Figure S1: Analytical hplc of thioether containing peptides **16** and **17**. Figure S2: Analytical hplc of Fmoc-Leu-alaninothiol prepared by coupling of Fmoc-Leu-OH on the Trt-resin **19** (m = 1; R′ = CH_3_) and subsequent cleavage by DTT (A) and TES (B). Figure S3: Analytical hplc (A) and ESI-MS analysis (B) of thiol-peptide **21** (ProTa (69-75) derivative), and hplc analytical hplc (C) and ESI-MS analysis (D) of thiol-peptide **22** (Hir (11-18) derivative), synthesized on Trt-resin **19** (R′ = CH_3_ (**21**); -CH_2_-Ph (**22**)). Figure S4: Analytical hplc of **26**. Figure S5: Analytical hplc during reaction of **27** and **28** at 2 h (a) and 24 h (b). The reaction process was monitored by following the peaks of the desired product **30** and the un-reacted **31** after their cleavage from resin; ESI-MS analysis of **30** (c).
