# Supplementary material for: Convergent Synthesis of Thioether Containing Peptides
Source: Molecules. 2020 Jan 5;25(1):218. doi: 10.3390/molecules25010218 (PMC6983143; doi:10.3390/molecules25010218)
Supplement: Supplementary file 1 [file molecules-25-00218-s001.pdf]

SUPPLEMENTARY DATA FOR:

Convergent Synthesis of Thioether Containing Peptides

Spyridon Mourtas [1,2], Christina Katakalou [1], Dimitrios Gatos [1] and Kleomenis Barlos [1,3,*]

[1] Department of Chemistry, University of Patras, 26510 Rio Patras, Greece; s.mourtas@upatras.gr (S.M.); c.katakalou@yahoo.gr (C.K.); d.gatos@upatras.gr (D.G.)

[2] Institute of Chemical Engineering Sciences of the Foundation for Research and Technology Hellas (FORTH/IEC-HT), 26504 Rio Patras, Greece

[3] CBL-Patras, Patras Industrial Area, Block 1, 25018 Patras, Greece.

* Correspondence: barlos@cblpatras.gr; Tel.: +30-2610-647600; Fax: +30-2610-647316.

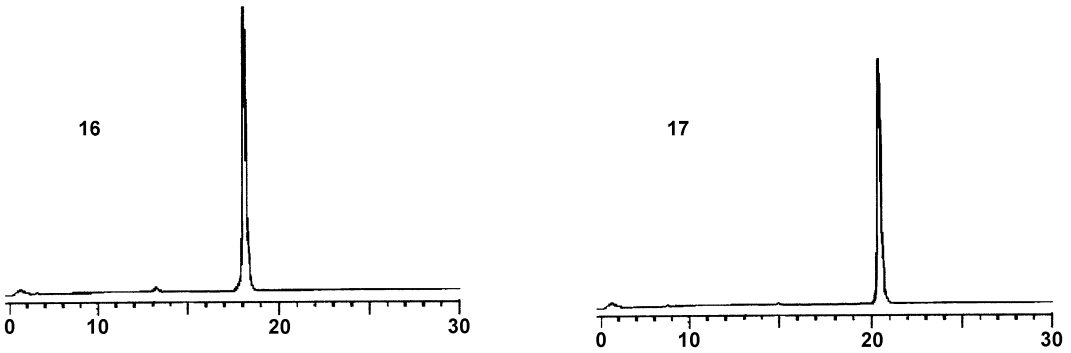

Figure S1. Analytical hplc of thioether containing peptides **16** and **17**; Column: Lichroshere RP-8, 5 μm, 4 x 150 mm; flow rate: 1 mL/min; gradient: from 20 to 100% acetonitrile in water within 30 min; detection at 265 nm.

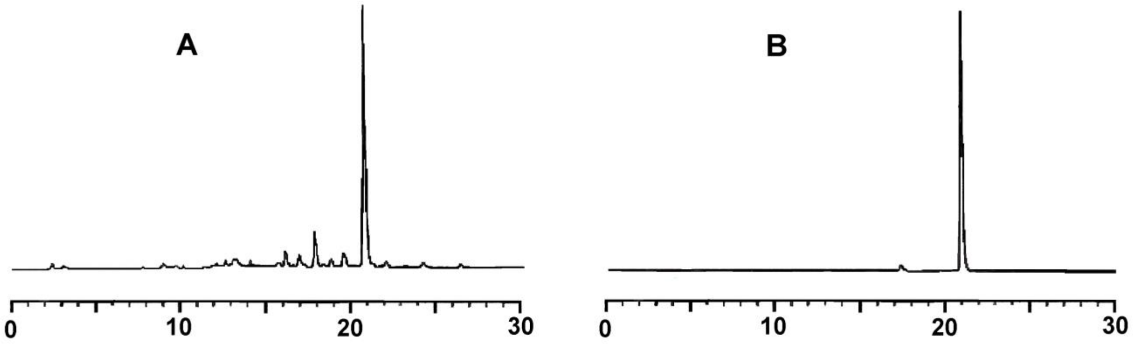

Figure S2. Analytical hplc of Fmoc-Leu-alaninothiol prepared by coupling of Fmoc-Leu-OH on the Trt-resin **19** (m = 1; R' = CH_3) and subsequent cleavage by DTT (A) and TES (B); Column: Nucleosil C8, 7 μm, 4 × 125 mm; flow rate: 1 mL/min; gradient: from 50 to 100% acetonitrile in water within 30 min; detection at 265 nm.

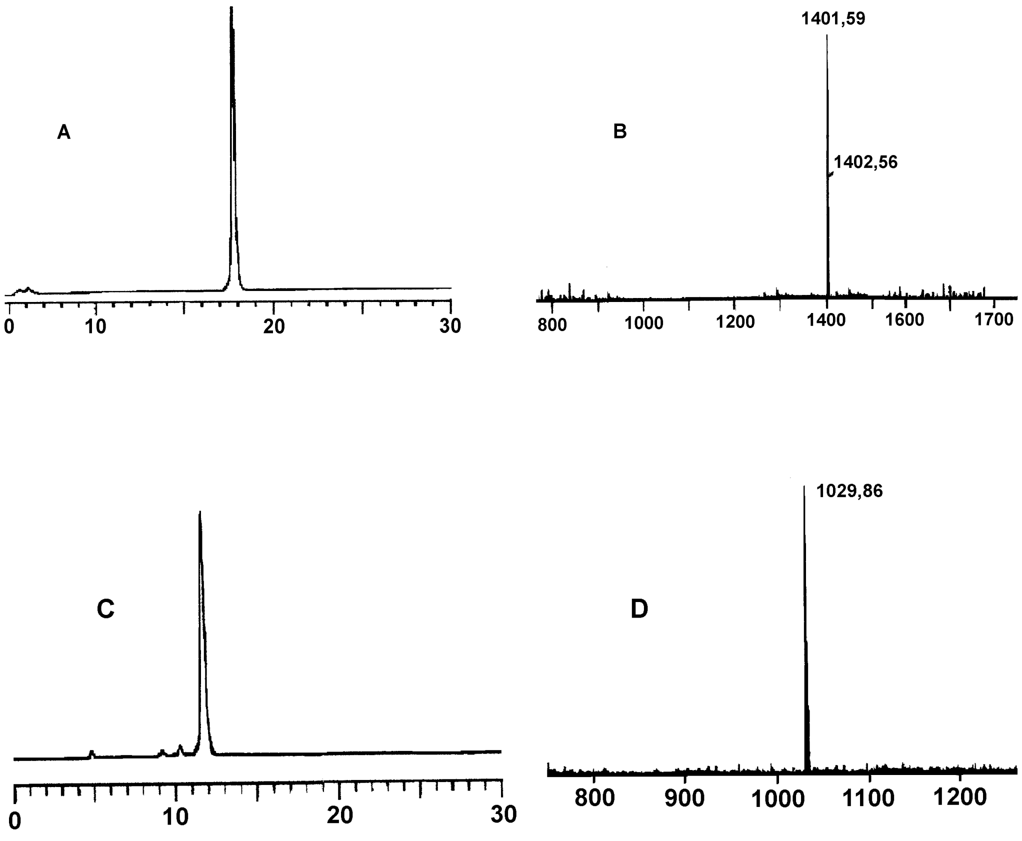

Figure S3. Analytical hplc (A) and ESI-MS analysis (B) of thiol-peptide **21** (ProTa (69-75) derivative), and analytical hplc (C) and ESI-MS analysis (D) of thiol-peptide **22** (Hir (11-18) derivative), synthesized on Trt-resin **19** (R' = CH_3 (**21**); -CH_2-Ph (**22**)); Column: Nucleosil C8, 7 μm, 4 × 125 mm; flow rate: 1 mL/min; gradient: from 50 to 100% acetonitrile in water within 30 min; detection at 265 nm.

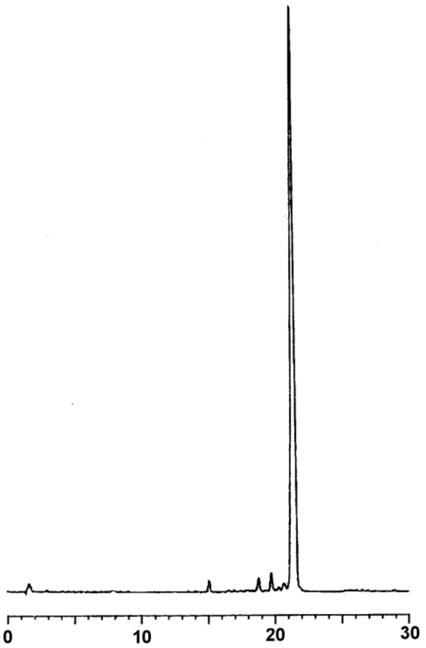

Figure S4. Analytical hplc of **26**; Column: Nucleosil C8, 7 μm, 4 × 125 mm; flow rate: 1 mL/min; gradient: from 50 to 100% acetonitrile in water within 30 min; detection at 265 nm.

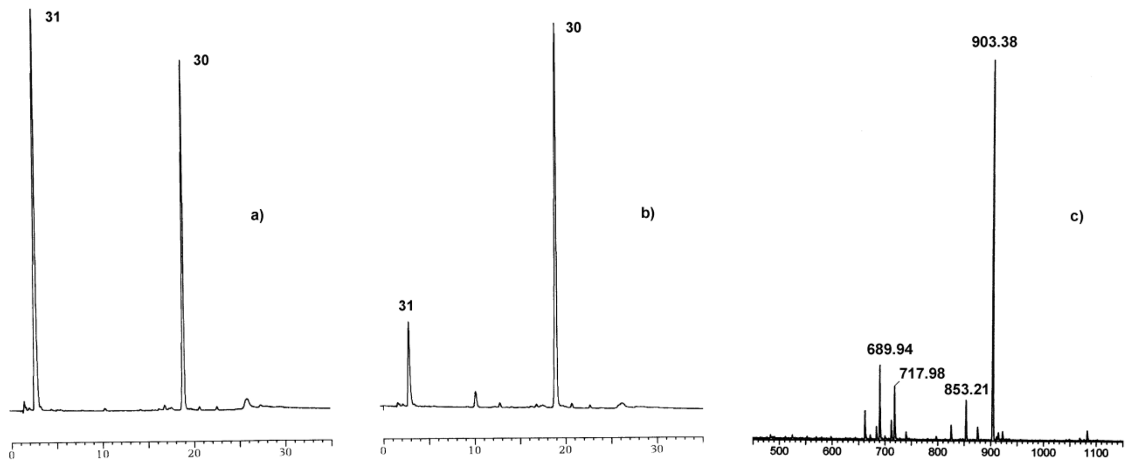

Figure S5. Analytical hplc during reaction of **27** and **28** at 2 h (a) and 24 h (b). The reaction process was monitored by following the peaks of the desired product **30** and the un-reacted **31** after their cleavage from resin; Column: Nucleosil C8, 7 μm, 4 × 125 mm; flow rate: 1 mL/min; gradient: from 50 to 100% acetonitrile in water within 30 min; detection at 265 nm; ESI-MS analysis of **30** (c).
